# Postpneumonectomy-like syndrome presenting in a patient with treated pulmonary tuberculosis: a case report

**DOI:** 10.1186/1752-1947-7-40

**Published:** 2013-02-12

**Authors:** Jennifer C Kam, Javier Dieguez, Vikram Doraiswamy, Enis Alberaqdar, Aparna Ramchandran, Marc Adelman, Alan J Klukowicz, Richard A Miller

**Affiliations:** 1 Seton Hall University School of Graduate Medical Education; St. Michael’s Medical Center, 111 Central Ave., Newark, NJ 07102, USA; 2 Division of Pulmonary and Critical Care Medicine, St Michael’s Medical Center, Seton Hall University, Newark, NJ, USA; 3 Department of Internal Medicine, St Michael’s Medical Center, Seton Hall University, Newark, NJ, USA

**Keywords:** Cor pulmonale, Dyspnea, Pneumonectomy, Postpneumonectomy-like syndrome, Postpneumonectomy syndrome, Pulmonary fibrosis, Tuberculosis

## Abstract

**Introduction:**

Postpneumonectomy syndrome is a rare condition that is characterized by dyspnea resulting from an extreme mediastinal shift and bronchial compression of the residual lung following surgical pneumonectomy. It is even rarer for this syndrome to present in patients without a history of prior lung surgery but induced by autopneumonectomy due to parenchymal disease, an entity termed ‘postpneumonectomy-like syndrome’.

**Case presentation:**

We present a rare case of a 91-year-old Puerto Rican man presenting with progressively worsening dyspnea with a history of pulmonary tuberculosis diagnosed 40 years earlier who developed severe unilateral lung fibrosis. Plain X-ray and computed tomography scans confirmed the presence of postpneumonectomy-like syndrome secondary to his parenchymal lung destruction. The patient developed cor pulmonale due to his extensive lung disease and as a consequence was not a suitable candidate for surgical intervention. The patient was otherwise stable until he developed acute respiratory distress from an acute upper gastrointestinal bleed and died four days into his hospital course.

**Conclusion:**

We present a rare case of postpneumonectomy-like syndrome as sequelae of severe pulmonary parenchymal tuberculosis infection along with a review of literature, in the hopes of aiding clinicians to include the differential of postpneumonectomy-like syndrome in patients presenting with worsening dyspnea without a history of surgical lung resection.

## Introduction

Postpneumonectomy-like syndrome is an uncommon complication of autopneumonectomy secondary to destructive lung disease [1-3]. Compensatory overexpansion of the contralateral lung results in mediastinal shifting towards the diseased lung, producing bronchial and possible tracheal compression. This phenomenon of mediastinal shifting has been documented following surgical lung resection, but rarely from disease-induced parenchymal lung destruction [1-5]. We report a case of postpneumonectomy-like syndrome as sequelae of a fibrotic lung from pulmonary tuberculosis. A review of this rare syndrome is included.

## Case presentation

A 91-year-old Puerto Rican man presented for evaluation of progressively worsening shortness of breath over the past six months associated with two weeks of productive cough of yellowish sputum. The patient also had complaints of fatigue, palpitations and dyspnea with minimal exertion. His past medical history was significant for pulmonary tuberculosis in 1969 for which he received approximately 24 months of medical treatment. The patient also had a history of coronary artery disease, bronchiectasis, moderate pulmonary hypertension, moderate chronic obstructive pulmonary disease (COPD), and has had multiple exacerbations of his bronchiectasis and COPD which did not require hospitalization. He had a history of smoking 30 cigarette packs per year but discontinued cigarette smoking 45 years prior to his admission. He denied alcohol or drug use and had no history of prior surgeries. He had been followed by a pulmonologist for over seven years, and spirometry testing done four months before his presentation showed an forced expiratory volume_1_ (FEV_1_) to forced vital capacity (FVC) ratio of 64%, FEV_1_ of 71%, and FVC of 84%. One year prior to presentation, an echocardiographic assessment demonstrated left ventricular hypertrophy with moderate pulmonary hypertension, and a dobutamine stress echocardiogram demonstrated a defect in the inferior wall consistent with a previous infarction.

**Figure 1:**
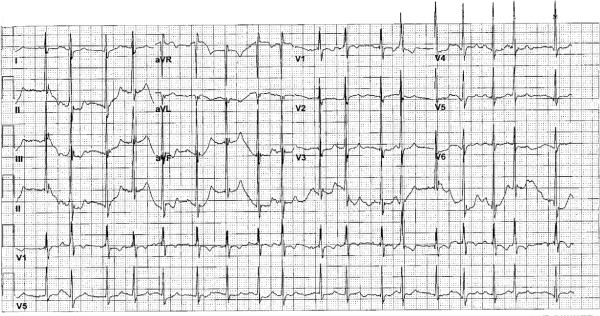
Multifocal atrial tachycardia without T wave or ST segment changes shown on 12-lead electrocardiogram.

**Figure 2:**
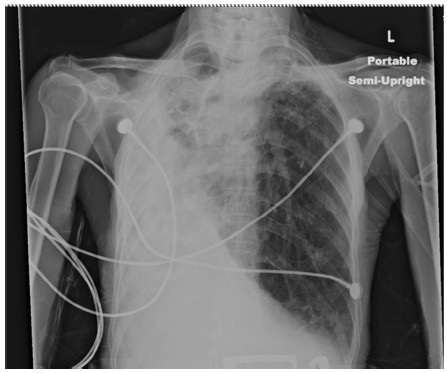
Chest radiograph showing gross mediastinal deviation toward the right lung and overexpansion of the left lung.

**Figure 3:**
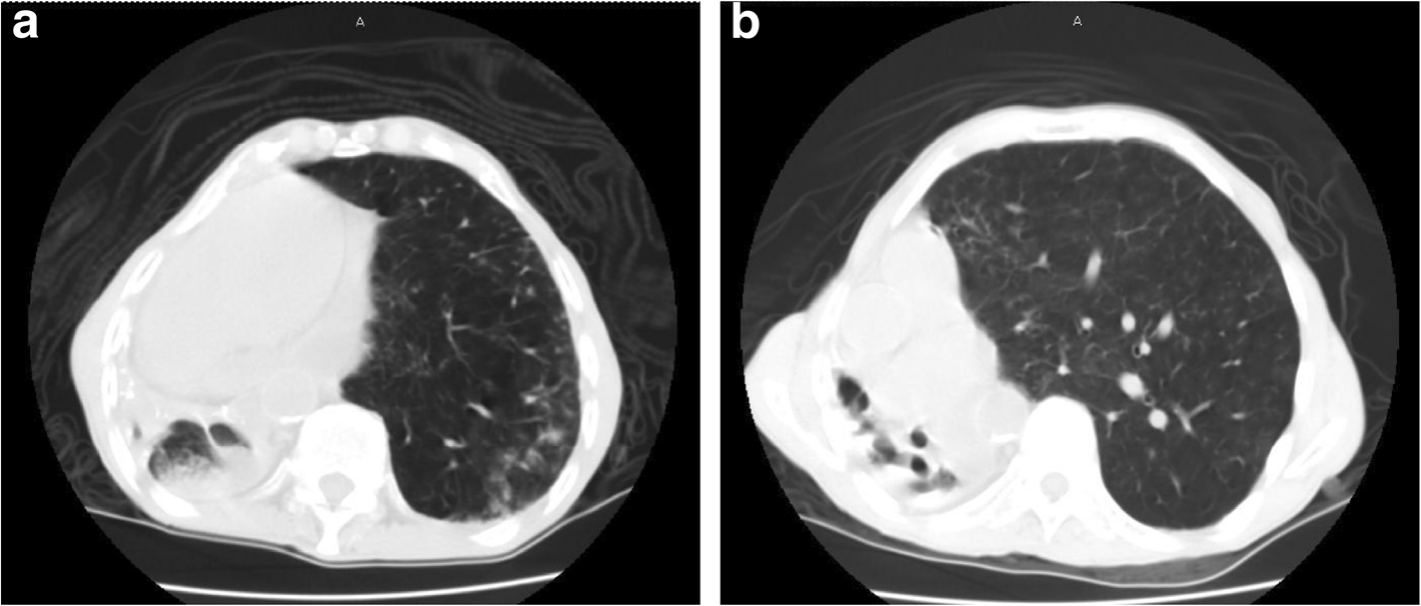
**(a) Computed tomography scan of the chest showing counterclockwise rotation of the mediastinum and great vessels. (b) **Computed tomography scan of the chest showing counterclockwise rotation of the mediastinum and great vessels; the trachea is to the right of the spine.

**Figure 4:**
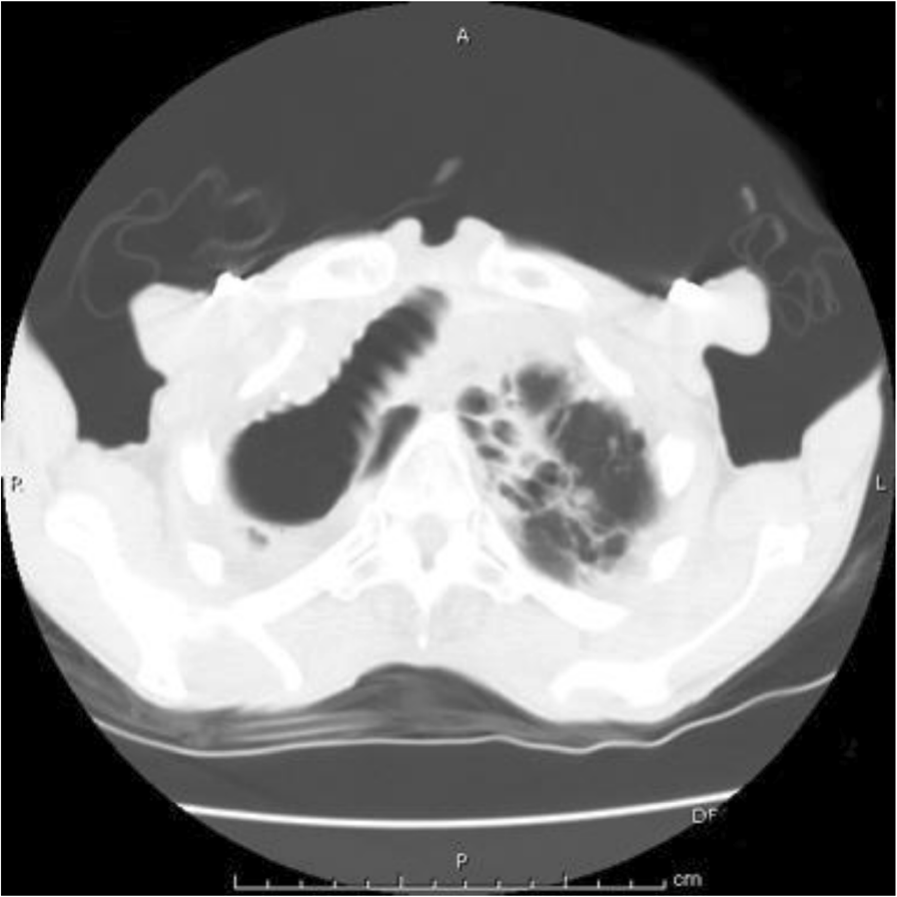
Computed tomography scan of the chest showing a dilated trachea shifted to the right hemithorax and left apical cystic bronchiectasis.

On physical examination, the patient was tachycardic at a heart rate of 140 beats per minute, tachypneic with a respiratory rate of 18 breaths per minute and saturating oxygen at 94% on atmospheric air. The patient was awake, alert, and oriented to person, time, and place and able to speak in full sentences. He did not demonstrate jugular venous distention, but his trachea was deviated towards his right side. There was no evidence of stridor. The patient’s breath sounds were diminished bilaterally with more prominently diminished sounds over his right lung fields. Coarse rhonchi were notable over his right lower lung. No wheezing or rales were appreciated. Heart sounds were more prominent on the right side of the chest and were characterized as an irregular tachycardia, with no murmurs, gallops or accentuated heart sounds appreciated. Laboratory studies demonstrated a white blood cell count of 7100/mm^3^, hemoglobin level 9.1g/dL, hematocrit 28.0%, sodium 137mEq/L, potassium 4.0mEq/L, chlorine 101mEq/L, bicarbonate 30mEq/L, blood urea nitrogen 16mg/dL, serum creatinine 0.6mg/dL, glucose 140mg/dL, troponin I: 0.03ng/mL, B-type natriuretic peptide 320ng/L, and normal liver and kidney function. Arterial blood gas showed a pH of 7.480, partial pressure of carbon dioxide of 46, partial pressure of oxygen of 116 and an oxygen saturation of 99% on two L of oxygen. An electrocardiogram showed a multifocal atrial tachycardia without T wave or ST segment changes (Figure 1). A chest X-ray demonstrated opacification of the right hemithorax with a marked mediastinal shift to the right and hyperinflation of the left lung (Figure 2). A computed tomography scan of the patient’s chest showed hyperinflation of his left lung, cystic bronchiectasis, mediastinal shifting to the right with clockwise rotation of his trachea and marked absence of normal lung parenchyma in his right lung (Figures 3, 4). Venous Doppler ultrasound of his lower extremities was negative for venous thrombosis. The patient’s presentation was attributed to a cardiac arrhythmia secondary to his extensive lung disease and was treated with calcium channel blockers to control his heart rate. However, four days into his hospital course, he became lethargic with a three gram drop of his hemoglobin and subsequently went into cardiac arrest. The patient was immediately intubated and was resuscitated according to Advanced Cardiac Life Support protocol. During the arrest he was noticed to have profound coffee ground emesis. Despite resuscitation efforts, the patient remained pulseless and subsequently died.

## Discussion

Postpneumonectomy syndrome is typically seen following surgical lung resection where the altered physiology ultimately leads to lung volume changes and exhibits as progressively worsening dyspnea [1-3]. Interestingly, postpneumonectomy syndrome is more commonly seen with right-sided lung resection, due to the counterclockwise rotation of the mediastinum into the pneumonectomy space [5]. When no surgical intervention is involved, a postpneumonectomy-like syndrome has been described where destructive lung disease is the etiological agent rather than surgical resection of the lung [4,5].

Pulmonary involvement of the infectious agent *Mycobacterium tuberculosis* can lead to bronchiectasis and, ultimately, pulmonary destruction and fibrosis if left untreated [5,6], as seen in our patient. Given the worldwide prevalence of pulmonary tuberculosis and its long-term complications, prevention of this progression relies on early detection and treatment of patients with pulmonary tuberculosis. However, in these patients 20% of chest radiographs are normal which therefore presents a diagnostic challenge for the clinician [6]. If diagnosed, treatment options for postpneumonectomy-like syndrome include not only treating the underlying cause but also repositioning the mediastinum with insertion of one or more tissue expanders [7].

Lee *et al.* (2008) described two case summaries where advanced tuberculosis led to unilateral lung destruction, resulting in postpneumonectomy-like syndrome [7]. In these cases, surgical correction using silicone-based tissue expanders in the hemithorax was necessary to reposition the severely deviated mediastinum and alleviate compression of the bronchi and trachea [8-10]. Realignment of the mediastinum resulted in marked improvement of dyspnea in both patients and their postoperative courses were unremarkable.

A similar presentation of postpneumonectomy-like syndrome, as a result of a non-infectious agent, has been described by Veronesi *et al.* (2002) [4]. In this case, the patient had severe fibrosis and complete atelectasis of the left lung as a complication of chemoradiation treatment for recurrent mediastinal Hodgkin’s lymphoma.

In the case we present, the right or left main bronchus was compressed between the main pulmonary artery and vertebra and aorta not by surgical pneumonectomy, but by autopneumonectomy that was secondary to tuberculosis. Surgical pneumonectomy is not a prerequisite for postpneumonectomy syndrome because this malady occurs as a result of stenosis of the bronchus after vigorous movement and rotation of the mediastinum. Postpneumonectomy-like syndrome must be considered in the differential diagnosis when patients exhibiting dyspnea have a tuberculosis-destroyed lung or a reduced lung volume that is caused by severe pulmonary fibrosis. Once this syndrome is diagnosed, mediastinal repositioning by insertion of one or more tissue expanders can be expected to lead to successful treatment.

## Conclusion

The features of postpneumonectomy syndrome typically present after a patient has undergone surgical lung resection and seldom occur secondary to a fibrotic lung. We present a rare case of a patient presenting with postpneumonectomy-like syndrome secondary to destructive pulmonary tuberculosis that resulted in a right-sided autopneumonectomy. This patient was not a candidate for surgical intervention due to his age and health status and, as a result, was managed conservatively.

## Consent

Written informed consent was obtained from the patient’s next of kin for publication of this case report and accompanying images. A copy of the written consent is available for review by the Editor-in-Chief of this journal.

## Competing interests

The authors declare that they have no competing interests.

## Authors’ contributions

JK has made substantial contributions to conception and design of the manuscript and was a major contributor in writing the manuscript. JD has made substantial contributions to conception and design of the manuscript, has been involved in revising it critically for important intellectual content and contributed in writing the manuscript. VD has been involved in drafting the manuscript and revising it critically for important intellectual content. AR has been involved in drafting the manuscript and revising it critically for important intellectual content. EA has been involved in drafting the manuscript and revising it critically for important intellectual content. MA, AK, RM made substantial contributions to conception and design of the manuscript and have been involved in revising it critically for important intellectual content. All authors read and approved the final manuscript.
